# Polyclonal Spread and Outbreaks with ESBL Positive Gentamicin Resistant *Klebsiella* spp. in the Region Kennemerland, The Netherlands

**DOI:** 10.1371/journal.pone.0101212

**Published:** 2014-06-27

**Authors:** Dennis Souverein, Stefan A. Boers, Dick Veenendaal, Sjoerd M. Euser, Jan Kluytmans, Jeroen W. Den Boer

**Affiliations:** 1 Department of Epidemiology and Infection Prevention, Regional Public Health Laboratory Kennemerland, Haarlem, The Netherlands; 2 Laboratory for Microbiology and Infection Control, Amphia Hospital, Breda, and VU University Medical Center, Amsterdam, The Netherlands; Institut Pasteur, France

## Abstract

**Objective:**

The objective of this study was to analyze the transmission dynamics of ESBL positive *Klebsiella* spp. with an additional resistance towards gentamicin (ESBL-G) in a Dutch region of 650,000 inhabitants in 2012.

**Methods:**

All patient related ESBL-G isolates isolated in 2012 were genotyped using both Amplification Fragment Length Polymorphism (AFLP) and High-throughput MultiLocus Sequence Typing (HiMLST). HiMLST was used to analyze the presence of (unidentified) clusters of ESBL-G positive patients. Furthermore, all consecutive ESBL-G isolates within patients were studied in order to evaluate the intra-patient variation of antibiotic phenotypes.

**Results:**

There were 38 ESBL-G isolates, which were classified into 18 different sequence types (STs) and into 21 different AFLP types. Within the STs, four clusters were detected from which two were unknown resulting in a transmission index of 0.27. An analysis of consecutive ESBL-G isolates (with similar STs) within patients showed that for 68.8% of the patients at least one isolate had a different consecutive antibiotic phenotype.

**Conclusion:**

The transmission of ESBL-G in the region Kennemerland in 2012 was polyclonal with several outbreaks (with a high level of epidemiological linkage). Furthermore, clustering by antibiotic phenotype characterization seems to be an inadequate approach in this setting. The routine practice of molecular typing of collected ESBL-G isolates may help to detect transmission in an early stage, which opens the possibility of a rapid response.

## Introduction

In Europe, there is an alarming increase in the prevalence of Multi Drug Resistant Organisms (MDROs) seen in recent years [1]. The increase of resistance in Gram-negative bacteria appears to be largely based on the production of Extended Spectrum Beta Lactamases (ESBLs) [2]. ESBL producing bacteria are able to deactivate the antibacterial properties of beta lactam antibiotics by hydrolysis [3]. In the Netherlands, the prevalence of ESBL producing *Klebsiella pneumoniae* increased from 3.3% in 2008, to 6.0% in 2012 [4]. The prevalence of patients cultured positive for ESBL positive *Klebsiella* spp. with an additional resistance towards gentamicin (ESBL-G) among all *Klebsiella* spp. culture positive patients in the region Kennemerland (650.000 inhabitants), was 2.6% in 2012 (unpublished data). Infections caused by ESBL-G isolates are not covered by the Dutch working party on antibiotic policy (SWAB), in which treatment with cephalosporins in combination with aminoglycosides (such as gentamicin) are advised as empirical therapy for sepsis [5]. Therefore, patients infected with ESBL-G are at risk for treatment failures, and as a consequence this infection is associated with higher morbidity, mortality and treatment costs compared to infections without ESBL-G [6]–[8]. For patients infected with an ESBL-G, ‘last-line antibiotics’ like intravenously applied carbapenems, are the preferred choice of treatment [6].

Between 1999 and 2011 several (small) outbreaks with Multidrug Resistant Klebsiella (MRK) were described in Dutch health care settings which comprised more than 100 patients [9]–[13]. Because patients-exchange regularly occurs between hospitals and nursing homes in the region Kennemerland, and the presence of ESBL-G positive patients can cause treatment failures, this finding has raised the following question: what are the transmission dynamics of ESBL-G and have there been (yet unidentified) clusters of ESBL-G in the region Kennemerland in 2012?

## Methods

### Ethics statement

According to the Dutch regulation for research with human subjects, neither medical or ethical approval was required to conduct the study since the data were retrospectively recorded. Additionally we received approval to conduct the study from the institutional review board of the Kennemer Gasthuis which waived the need for participant consent. The data were anonymized and analyzed under code.

### Study design and bacterial isolates

To answer the main question, all patient related ESBL-G isolates (from January 2012 to December 2012), which were routinely collected by the Regional Public Health Laboratory Kennemerland (RPHLK), were retrospectively included in our study. We genotyped all ESBL-G isolates by using both Amplification Fragment Length Polymorphism (AFLP) and High-throughput Multilocus Sequence Typing (HiMLST). Subsequently, we analyzed the spread and possible presence of (yet unidentified) clusters of ESBL-G positive patients that could be detected by applying these two different genotyping techniques. The data were analyzed with regard to admission dates and in house location of the ESBL-G positive patients. Furthermore, we compared the results of the HiMLST, AFLP and a phenotypical method based on species type and antibiogram to determine their discriminatory capacity. Additionally, we analyzed all consecutive ESBL-G isolates (with similar sequence types (STs)) within patients to evaluate the intra-patient variation of phenotypes based on the antibiogram. The RPHLK stored all first isolated ESBL positive isolates (phenotypic distinctive, per sampling date) per patient in the freezer for future analysis, allowing the possibility to conduct this study. In total 177 isolates were found to be ESBL-G positive. Of these 177 isolates 75 isolates were excluded for various reasons: 20 isolates were not patient related, 40 isolates were marked as a double isolate (identical phenotype from the same patient) and therefore not stored in the freezer. For five isolates the AFLP and/or HiMLST was not reliable due to mixed reads. This resulted in 102 isolates with complete antibiogram and genotyping results. Among these 102 isolates, only the first positive isolate per patient was included resulting in 38 patient related ESBL positive *Klebsiella* spp. isolates which were analyzed. Additionally, we considered 76 consecutive ESBL-G isolates (with similar STs) within patients to analyze the intra-patient variation of phenotypes. The RPHLK performs microbiology for all (three) hospitals, most general practitioners and most nursing homes in the region Kennemerland which comprise over 650.000 inhabitants. Hospital one is a 260 bed regional hospital, hospital two and three are 500 bed teaching hospitals.

### Microbiological methods

All isolates were tested for resistance patterns using the Vitek2 System (BioMérieux). Isolates suspected for ESBL production (lowered susceptibility for ceftazidime and/or cefotaxime) were further determined using the matrix-assisted laser desorption/ionization time-of-flight mass spectrometry (MALDI-TOF-MS) (Bruker Daltonics, Germany). Phenotypic confirmation of ESBL was performed using the combination disk method using cefotaxim and ceftazidime with and without clavulanic acid (Becton Dickinson, Netherlands). All tests were performed and interpreted according to the National Guideline for Laboratory Detection of ESBL [14].

### Molecular typing by Amplified Fragment Length Polymorphism

Dna-lysates of ESBL positive *Klebsiella* spp. isolates were genotyped by Amplified Fragment Length Polymorphism (AFLP) using the restriction enzymes *Eco*RI and *Mse*I according to previously described methods [15]. Digested-ligated products were amplified with adaptor-specific primers with selective extensions, *Mse*+C and *Eco*RI+A. Primer *Eco*RI+A was labeled with D3 for fragment separation with the CEQ8000 Genetic Analysis System (Beckman-Coulter). The collected raw data were analyzed using the Bionumerics v6.6 software (Applied Math). To analyze and group AFLP patterns a Pearson correlation UPGMA with a curve smoothing of 0.5%, and an optimization of 2% was used. Isolates were considered clonally related when *Klebsiella* strains were identical, including strains with a concordance higher than >90%.

### Molecular typing by High-throughput MultiLocus Sequence Typing

All the available *Klebsiella* spp. isolates were subjected to MLST. For this, partial DNA sequences of the seven housekeeping genes *gap*A, *inf*B, *mdh, pgi*, *pho*E, *rpo*B and *ton*B were generated using the High-Throughput-MultiLocus Sequence Typing (HiMLST) strategy as described by Boers et al [16]. The oligonucleotides used for PCR amplification reported in the standardized MLST scheme by Diancourt et al were modified to reduce amplicon sizes and to contain universal tails for the employment of HiMLST (Table S1) [17]. Allele variant numbers and corresponding sequence types (STs) were obtained by performing queries in the *Klebsiella* MLST database, which is available online [18].

### Antibiotic phenotype

The antibiotic phenotype characterization of the isolates was based on species type (*Klebsiella pneumoniae* or *Klebsiella oxytoca*) combined with a number of selected antibiotics: ciprofloxacin-co trimoxazol-tobramycin-carbapenem-nitrofurantoin. Resistance towards carbapenem was defined as resistance towards either (or both) meropenem and imipenem.

### Definition of ESBL-G clusters, epidemiological linkage and transmission index

A cluster of ESBL-G was defined as two or more patients with identical Sequence Types (STs) and epidemiological linkage, which was defined as patients who had stayed on the same ward in the same hospital or primary care institution within a maximum time window of four weeks [19]. The transmission index was calculated as the number of secondary cases (number of patients with epidemiological linkage, without the index patient) divided by the number of index patients plus the number of primary cases (single patients without clustering).

### Intra-patient (antibiotic phenotypic) comparison of consecutive ESBL-G isolates

All consecutive isolates (with equal ST as the first isolate) of ESBL-G positive patients were compared with the first isolate and included in these analyses. A consecutive isolate was marked as different when resistance towards one of the included antibiotics changed (as an example: from resistant to susceptible or the other way around).

## Results

### Demographic and clinical characteristics of ESBL-G carriers

In 2012, 38 patients were diagnosed with an ESBL positive gentamicin resistant *Klebsiella* spp. isolate in the region Kennemerland, the Netherlands (Table 1). The mean (SD) age of these patients was 66.5 (18.8) years, and 19 (50%) patients were male. Sixteen (42.1%) patients were diagnosed in the primary care setting, ten (26.3%) patients in hospital 2, and six (15.8%) patients in both hospital 1 and 3. The isolates were derived from a broad range of non-sterile body sites (see Table 1). The majority of isolates were determined as *Klebsiella pneumoniae* (34 isolates (89.5%)) followed by *Klebsiella oxytoca* (four isolates (10.5%)). All isolated *Klebsiella oxytoca* isolates were diagnosed in hospital 1.

**Table 1:** Demographic and clinical characteristics of patients and isolates.

Patient characteristics	Total	Hospital 1	Hospital 2	Hospital 3	Primary care
**Number of isolates and patients**	38 (100)	6 (15.8)	10 (26.3)	6 (15.8)	16 (42.1)
**Gender**					
** Male**	19 (50)	3 (50)	6 (60)	5 (83.3)	5 (31.3)
**Mean age, yrs (SD)**	66.5 (18.8)	69.3 (9.9)	66.2 (21.1)	67.7 (10.0)	65.3 (23.2)
**Sample sites**					
**Non sterile**					
** Gastro-intestinal tract** †	11 (28.9)	1 (16.7)	4 (40)	1 (16.7)	5 (31.2)
** Catheter**	1 (2.6)	0	0	1 (16.7)	0
** Throat**	1 (2.6)	0	1 (10)	0	0
** Sputum**	3 (7.9)	2 (33.3)	1 (10)	0	0
** Urine**	9 (23.8)	0	1 (10)	2 (33.3)	6 (37.5)
** Urine catheter**	7 (18.5)	1 (16.7)	1 (10)	1 (16.7)	4 (25)
** Wound**	4 (10.5)	2 (33.3)	1 (10)	0	1 (6.3)
** Other**	1 (2.6)	0	1 (10)	0	0
**Sterile**					
** Blood**	1 (2.6)	0	0	1 (16.6)	0
**Species**					
*** Klebsiella*** ** pneumoniae**	34 (89.5)	2 (33.3)	10 (100)	6 (100)	16 (100)
*** Klebsiella*** ** oxytoca**	4 (10.5)	4 (66.7)	0	0	0

† including faeces, perineum, rectum and peri-anal samples.

Data are presented as numbers (%) unless indicated otherwise.

### HiMLST, AFLP and phenotypical method

The results of the molecular and phenotypical analyses are shown in Table 2. The isolates were classified into 18 different STs and 21 different AFLP types. The phenotypical analyses consisting of species type and sensitivity patterns for a selection of antibiotics (ciprofloxacin-co trimoxazol-tobramycin-carbapenem-nitrofurantoin) classified the isolates into 17 different antibiotic phenotypes. As displayed in Table 2, the AFLP types showed similar results in comparison with HiMLST, except for ST 405 (two different AFLP types, type M and type J), ST 37 (two different AFLP types, type I3 and K) and ST 17 (three different AFLP types, type L1, L2 and L3). These discrepancies resulted in a concordance between typing techniques of 84.6% (number of different AFLP types divided by the number of isolates of which two of more STs were available).

**Table 2:** All first isolated patient related strains with corresponding sequence type, AFLP type and phenotype.

Strain number †	HiMLST	AFLP	Phenotype *	Location of diagnosis	Cluster (HiMLST) ‡	Cluster (AFLP) ¥
1	147	N	KP-RRISI	Primary care	–	–
2	161	I2	KP-RRISR	Primary care	–	–
3	17	L1	KP-IRRSI	Primary care	–	–
4	17	L1	KP-IRRSI	Hospital 3	–	–
5	17	L1	KP-RRRSR	Primary care/Hospital 3	–	–
6	17	L2	KP-IRRSS	Hospital 3	–	–
7	17	L3	KP-IRRSI	Hospital 1	–	–
8	17	L3	KP-RRRSR	Primary care	–	–
9	193	R	KP-RRRSI	Hospital 2	A	A
10	193	R	KP-RRRSI	Primary care	–	–
11	193	R	KP-RRRSR	Hospital 2	A	A
12	37	I3	KP-SRISR	Hospital 3	–	–
13	37	I3	KP-SRISR	Primary care	–	–
14	37	K	KP-RRRSR	Hospital 1	–	–
15	392	P	KP-RRRRS	Hospital 3	–	–
16	405	J	KP-IRRSR	Hospital 2	B	–
17	405	M	KP-IRISI	Hospital 2	–	–
18	405	M	KP-IRISI	Primary care/Hospital 2	–	–
19	405	M	KP-IRRSR	Hospital 2	–	–
20	405	M	KP-IRRSR	Hospital 2	B	B
21	405	M	KP-RRISR	Primary care/Hospital 2	–	–
22	405	M	KP-RRRSR	Hospital 2	B	B
23	405	M	KP-RRRSR	Hospital 2	B	B
24	414	H	KP-SRSSI	Hospital 2	–	–
25	45	I4	KP-SRISI	Primary care	–	–
26	641	W	KP-SSISI	Hospital 3	–	–
27	946	I5	KP-IRRSR	Primary care	–	–
28	KO_01	A	KO-RRRSI	Hospital 1	C	C
29	KO_01	A	KO-RRRSS	Hospital 1	C	C
30	KO_01	A	KO-RRRSS	Hospital 1	C	C
31	KO_02	B	KO-SRRSI	Hospital 1	–	–
32	1418	I1	KP-SRSRR	Primary care	–	–
33	1420	U	KP-RRISR	Hospital 3	–	–
34	1421	E1	KP-IRRSS	Hospital 2	–	–
35	1423	X	KP-IRRSS	Primary care	–	–
36	1207	I4	KP-RRRSI	Primary care (nursing home A)	D	D
37	1207	I4	KP-RRRSR	Primary care (nursing home A)	D	D
38	1207	I4	KP-RRRSR	Primary care (nursing home A)	D	D

HiMLST = High-troughput multilocus sequence typing.

AFLP = Amplification Fragment Length Polymorphism.

*Phenotype = Species type and resistance patterns for ciprofloxacin-co trimoxazol-tobramycin-carbapenem-nitrofurantoin. S = susceptible I = intermediate R = resistant.

‡A cluster of ESBL-G was defined as two or more patients with epidemiological linkage and the same ST-type.

†Only the first positive isolate per patient is included.

¥A cluster of ESBL-G was defined as two or more patients with epidemiological linkage and the same AFLP-type.

The phenotypical characterization showed a high variation between isolates with the same sequence type and AFLP type. All STs (with more than one strain) showed two or more different antibiotic phenotypes.

### Clustering and transmission

**Figure 1:**
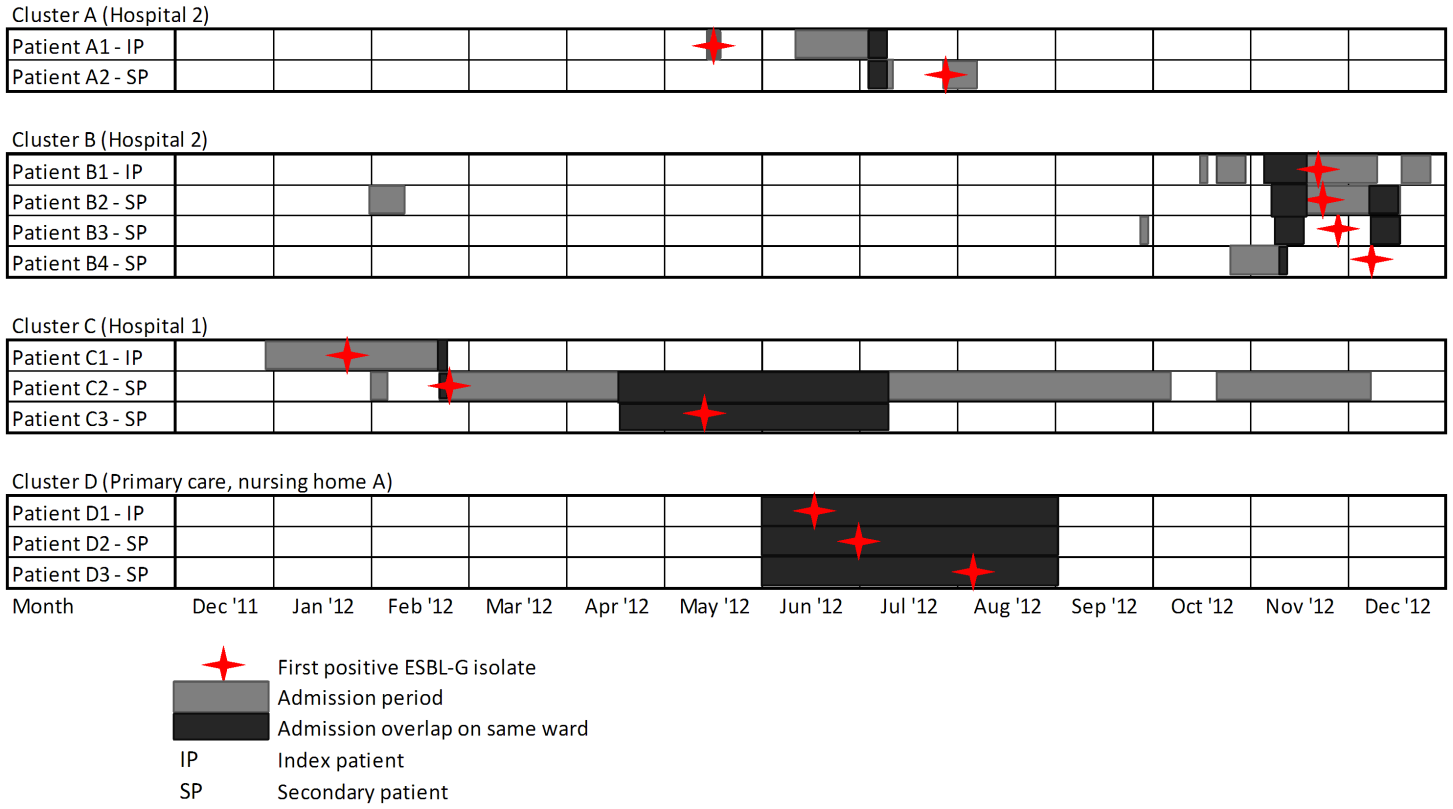
Transmission dynamics of ESBL positive *Klebsiella* strains with an additional resistance towards gentamicin (ESBL-G) in 2012. Grey boxes correspond with admission periods for each patient. The star symbol marks the date of first ESBL-G positive culture. Black boxes represent the periods of overlap in admission time and ward between patients. IP represents index patients and SP represents secondary patients according to the definitions.

Based on sequence type (obtained from the HiMLST analysis) and clustering definitions, we could differentiate four clusters (Figure 1). Three clusters were detected in the hospitals, and one cluster was detected in a nursing home (Table 2). No transmission was detected between hospitals and none of the patients was transferred to another hospital. The largest cluster comprised four patients colonized/infected with ST 405 located in hospital 2 (cluster B). Furthermore, we identified three other clusters: ST 193 (cluster A, hospital 2), KO_01 (cluster C, hospital 3) and ST 1207 (cluster D, primary care). Based on AFLP typing, we could differentiate the same four clusters that were detected with HiMLST (Cluster A–D). Instead of the four patients in cluster B who were identified by HiMLST, AFLP typing identified only three of these patients.

Three patients of cluster B (patient B1, B2 and B3, hospital 2) were part of a known cluster. We additionally linked one more patient (patient B4 ) to this cluster based on the HiMLST analyses performed in this study. All patients in cluster B were diagnosed between November 2012 and December 2012. After patients B1, B2, and B3 were identified and an ESBL-G isolate was isolated from a siphon (located in the room of the colonized patients) infection prevention procedures consisting of contact isolation on a single room (following the national directive for MDRO) and replacement of the siphon successfully stopped transmission.

The patients of cluster C (hospital 1) were also known before the start of this study. All patients were diagnosed between January 2012 and May 2012. After infection prevention procedures, consisting of contact isolation on a single room were installed (following the national directive for MDRO), transmission was stopped successfully.

Additionally, two new clusters (A and D) were identified after the molecular analysis performed in this study. Cluster A was detected in hospital 2 and consisted of two patients (diagnosed between May 2012 and July 2012). Cluster D comprised three patients living in a nursing home which were linked based on strain typing results (diagnosed between June 2012 and August 2012). Without this study these clusters were not identified. However all patients were isolated following the national directive for MDROs. In total, eight patients acquired ESBL-G as a result of transmission (following the criteria of epidemiological linkage and clustering), while four patients were classified as index patients. 26 patients were classified as primary cases. The overall transmission index of ESBL-G in the region Kennemerland in 2012 was 0.27.

### Intra-patient comparison of phenotypes

Sixteen of the 38 ESBL-G positive patients had more than one consecutive ESBL-G isolate (42.1%) with an identical sequence type as the first isolate (all first isolates shown in Table 2) available. After analyzing all 76 consecutive ESBL-G isolates of these sixteen patients, the data showed that eleven patients (68.8%) had at least one different consecutive antibiotic phenotype.

## Discussion

For this present study, we genotyped all isolated ESBL positive *Klebsiella* spp. with an additional resistance towards gentamicin (ESBL-G) derived from patients in the region Kennemerland, the Netherlands in 2012, by using AFLP, HiMLST and an antibiotic phenotypical method. Although the prevalence of ESBL-G positive patients among all *Klebsiella* spp. positive patients was relatively low (2.6% in 2012), several clusters were identified. By genotyping (using AFLP and HiMLST) all ESBL-G strains, we detected four clusters, of which two were previously unknown. No transmission between institutions was detected. A possible explanation for this finding is that none of the positive patients was transferred between hospitals. The genotyping data showed comparable results for AFLP and HiMLST: both techniques identified four clusters. Only in cluster B one patient was missed when only AFLP was used. Seen from an epidemiological point of view this patient is correctly classified as part of cluster B since there was an epidemiological link present (admission on same ward in a time window of 4 weeks). Although we found some discrepancies between AFLP and HiMLST (concordance of 84.6%) it is difficult to explain the cause of these discrepancies as most of these patients had no epidemiological link.

The phenotypical method, consisting of the identification of species type and resistance towards several selected antibiotics, could not adequately detect these clusters. These data suggest that, in this setting, the phenotypical method (using an antibiogram) is not suitable for the identification of clusters among ESBL-G isolates. However, the antibiotic susceptibility testing does provide relevant information with respect to the treatment of patients, as the different ESBL-G strains isolated within patients showed high variation in the antibiogram.

Although several studies have described outbreaks of multiresistant *Klebsiella* spp. (MRK) including ESBL-G, this study is (to our knowledge) the first Dutch study showing regional transmission dynamics of ESBL-G in both hospitals and primary care patients [9]–[13]. In 2011, the TRIANGLE study described the transmission of highly resistant gram-negative microorganisms including MRK in 18 Dutch hospitals by analyzing routine clinical samples during a six month period [13]. The same study showed low horizontal transmission rates (ranging from 0.0 to 0.2) and detected 22 clusters (in 18 hospitals) by using AFLP. Most of the isolated enterobacteriaceae (54.3%) were ESBL producers. In the present study we found a transmission rate of 0.27. Although this rate is not directly comparable with the results of the previous mentioned study (because different bacterial species were studied) it does indicate a considerable transmission capacity of ESBL-G *Klebsiella* spp. A comparison between other studies is difficult because different definitions, other bacterial species and/or single centers were studied.

When analyzing the consecutive ESBL-G *Klebsiella* isolates (with identical STs) within patients, we found a high percentage of variation in antibiogram of these intra-patient consecutive isolates. A possible explanation for the variation in antibiograms is that the genes encoding for resistance of these antibiotics (especially aminoglycosides and quinolones) are frequently found on plasmids, and could be selected out by the use of these antibiotics in the treatment protocol of the patients. Several previous studies have reported this plasmid mediated co-resistance in ESBL positive bacteria [20], [21].

Genotyping MRK can be performed using different techniques. These techniques contain fragment based methods such as: AFLP, PFGE, Rep-PCR and MLVA but also DNA sequence techniques such as MLST [22], [23]. In this study, we used AFLP and HiMLST to genotype ESBL-G isolates. One of the advantages of using AFLP is the faster procedure time, which may be essential for genotyping in local epidemiological and outbreak investigations. Furthermore, with AFLP almost the whole genome is covered, resulting in a higher discriminatory capacity [24]. A possible drawback of the AFLP technique is the absence of an inter-laboratory database and the low inter-laboratory reproducibility of this technique, caused by the different platforms that are used worldwide. As a result, no comparison in global epidemiology is possible with the results of the AFLP typing data. On the other hand MLST uses an internationally accepted nomenclature, targeting seven housekeeping genes regardless of the platform used to generate them, showing its comparability [24]. In term of costs and labor, MLST is much more labor intensive and expensive than the AFLP technique. However, the introducing of HiMLST has resulted in a sharp reduction of costs since this method allows the simultaneous analysis of a large number of isolates. By using HiMLST, the price per analyzed isolate is comparable to that of AFLP [16]. Considering these arguments, HiMLST seems to be the most suitable technique for regional monitoring procedures including genotyping.

Clustering patients on the basis of equal STs (without an epidemiological link) can be interpreted in several ways. (1) The appearance of the strain could simply reflect polyclonal spread rather than transmission [25]. (2) On the other hand transmission could be present, but not identified since carriership is generally asymptomatic [26]. As a result, intermediate patients are missed and no epidemiological link can be made. However, when patients with ESBL-G isolates with identical STs in addition show epidemiological linkage, transmission is probably the case. One must be careful to conclude there is no transmission, taking into account the possibility of asymptomatic carriership. Nevertheless, in our study the difference between a time window of one day or four weeks in the epidemiological link definition did not increase the number of secondary cases.

The present study has several limitations. First of all, no plasmid typing was performed on the collected ESBL-G isolates. Since we described regional transmission of ESBL-G, it would be interesting to assess ESBL producing genes, as plasmid transmission is possible between bacteria of the same and other species [27]. This could possibly clarify transmission routes, or help to identify yet unknown reservoirs. Second, we have retrospectively described the regional transmission on the basis of clinical samples, collected from symptomatic patients which is a major drawback of this study. As it is well known that colonization of ESBL positive bacteria is not uncommon among the hospitalized population, the extent of the transmission could be much larger than described, since colonization could be established without infection [28]. For future studies we would advice using a prospective study design, including for example screening of all contact patients when an ESBL-G positive patient is detected (in clinical samples). This prospective study design requires a lot of cooperation and effort to perform regionally, especially in all participating nursing homes.

In conclusion, our results show that the transmission of ESBL-G *Klebsiella* in the region Kennemerland is polyclonal (without transmission between institutions) with several outbreaks (with the majority of patients being part of clusters with a high level of epidemiological linkage) that could be identified. Furthermore, clustering by antibiogram phenotype characterization seems to be an inadequate method in this setting. The routine practice of molecular typing of collected ESBL-G isolates may help to detect nosocomial spread in an early stage, which opens the possibility of a rapid response.

## Supporting Information

Table S1 **MLST target gene-specific primers used in this study.** Nucleotides in black represent the gene-specific part and universal tails are shown in red or blue.
(DOC)
