# Supplementary material for: Polyclonal Spread and Outbreaks with ESBL Positive Gentamicin Resistant Klebsiella spp. in the Region Kennemerland, The Netherlands
Source: PLoS One. 2014 Jun 27;9(6):e101212. doi: 10.1371/journal.pone.0101212 (PMC4074119; doi:10.1371/journal.pone.0101212)
Supplement: Table S1 — MLST target gene-specific primers used in this study. Nucleotides in black represent the gene-specific part and universal tails are shown in red or blue. (DOC) [file pone.0101212.s001.doc]

**Table S1. MLST target gene-specific primers used in this study.** Nucleotides in black represent the gene-specific part and universal tails are shown in red or blue.

| **Locus** | **Direction** | **Primer sequence (5’ to 3’)** |
| --- | --- | --- |
| gapA_F | Forward | GACACTATAGTGAAATATGACTCCACTCACGG |
| gapA_R | Reverse | CACTATAGGGCTTCAGAAGCGGCTTTGATGGCTT |
| infB_F | Forward | GACACTATAGCTCGCTGCTGGACTATATTCG |
| infB_R | Reverse | CACTATAGGGCGCTTTCAGCTCAAGAACTTC |
| mdh_F3 | Forward | GACACTATAGTCATATCCCCACAGATG |
| mdh_R2 | Reverse | CACTATAGGGCTGCATCGCGCGAACCAG |
| pgi_F | Forward | GACACTATAGAGAAAAACCTGCCTGTACTGCTGGC |
| pgi_R3 | Reverse | CACTATAGGGTCGAAGGTGAAGATGTTG |
| phoE_F | Forward | GACACTATAGACCTACCGCAACACCGACTTCTTCGG |
| phoE_R | Reverse | CACTATAGGGTGATCAGAACTGGTAGGTGAT |
| rhoB_F2 | Forward | GACACTATAGCTTCGAAGTTCGAGACGTAC |
| rhoB_R2 | Reverse | CACTATAGGGCATCCACGTACTGAACG |
| tonB_F | Forward | GACACTATAGCTTTATACCTCGGTACATCAGGTT |
| tonB_R | Reverse | CACTATAGGGATTCGCCGGCTGRGCRGAGAG |
